# To Prevent Strangling during Extraction

**Published:** 1909-03-15

**Authors:** F. H. Skinner

**Affiliations:** Chicago


### TO PREVENT STRANGLING DURING EXTRACTION.

F. H. SKINNER, D.D.S., CHICAGO.

When extracting a tooth or doing a surgical operation in the mouth, under a general anaesthetic, just as the patient is fairly well under, put the finger on base of tongue, bring it forward and place a large roll of cotton, two or three inches long, across the base, just forward of the epiglottis; the tongue will not drop back and no blood or debris will not get back of cotton. If the operation is a long one, change cotton as often as necessary by drawing one end of roll forward and passing fresh one into place. If this is done until patient has regained consciousness sufficiently to expectorate, no blood will trickle down the throat to be thrown up afterward, or worse still, get into the trachea to cause strangulation. Nor is there any danger of a tooth or root slipping out of the forceps and going down the throat thereby causing the operator to suddenly develop a case of nervous prostration.

